# School Burnout Inventory: Factorial Validity, Reliability, and Measurement Invariance in a Chilean Sample of High School Students

**DOI:** 10.3389/fpsyg.2021.774703

**Published:** 2022-01-04

**Authors:** Marcos Carmona-Halty, Patricio Mena-Chamorro, Geraldy Sepúlveda-Páez, Rodrigo Ferrer-Urbina

**Affiliations:** Escuela de Psicología y Filosofía, Universidad de Tarapacá, Arica, Chile

**Keywords:** school burnout, psychometric analyses, high school students, Chilean students, gender invariance

## Abstract

This brief report assessed the psychometric validity and gender invariance of the School Burnout Inventory (SBI) –a measure of students’ exhaustion, cynicism, and inadequacy– in a convenience sample of 972 high school Chilean students ranging between 12 and 18 years old. The results showed that: (1) the SBI produces adequate scores in terms of reliability; (2) two models (one solution of three related factors and one of second-order and three first-order factors) fitted adequately fit to our sample and was invariant across gender; and (3) the SBI scores were significantly related to other related constructs (i.e., study-related emotions, academic psychological capital, and academic engagement). Overall, the SBI was found to be a reliable and valid inventory to assess school burnout in Chilean high school students.

## Introduction

The term burnout was first used in the late 70s to describe a state of exhaustion associated with human services professionals (Maslach and Jackson, 1981; Maslach et al., 2001; Schaufeli et al., 2002; Sakakibara et al., 2020). However, in the following years, this construct has become a social and scientific topic of great attention due to its extension to other domains, including students, and its impact on mental and physical health, achievement, and subsequent academic career (Fiorilli et al., 2014; Walburg, 2014; Walburg et al., 2016; Gabola et al., 2021). In this line, *school burnout* describes those students’ who experience *emotional exhaustion* –characterized by feelings of strain and chronic fatigue resulting from overtaxing schoolwork, *cynicism* –characterized by an indifferent or a distal attitude toward schoolwork in general, a loss of interest in one’s academic work, and not seeing it as meaningful, and *sense of inadequacy* –diminished feelings of competence and less achievement, and lack of accomplishment both in one’s schoolwork and in school as a whole (Kiuru et al., 2008; Salmela-Aro et al., 2008, 2009b; Salmela-Aro and Upadyaya, 2014; Salmela-Aro, 2016; Salmela-Aro and Read, 2017).

Currently, we know that school burnout is directly related to school dropout (Bask and Salmela-Aro, 2013), boredom and neuroticism (Sulea et al., 2015), academic anxiety (Fiorilli et al., 2020), depressive symptoms (Moyano and Riaño-Hernández, 2013; Ponkosonsirilert et al., 2020), and suicidal ideas (Walburg et al., 2016). Conversely, it is inversely related to schoolwork engagement (Bresó et al., 2011; Salmela-Aro and Upadyaya, 2012), basic psychological needs (Sulea et al., 2015), well-being (Raiziene et al., 2013), and achievement (May et al., 2015; Gungor, 2019). Also, it has been found, based on the demands-resources model (Demerouti et al., 2001; Schaufeli and Bakker, 2004; Bakker and Demerouti, 2007), that study demands (e.g., schoolwork overload), and study resources (e.g., hope, efficacy, resilience, optimism) promote and prevent their appearance, respectively (Salanova et al., 2010; Salmela-Aro and Upadyaya, 2014; Gungor, 2019; Romano et al., 2021).

Research on the field has mostly been carried out using the School Burnout Inventory (SBI) developed by Salmela-Aro et al. (2009a). The SBI was initially validated in a Finnish context, demonstrating that school burnout can be explained both as a solution of three correlated factors (i.e., exhaustion, cynicism, and inadequacy) and by a second-order structure (i.e., school burnout) and three first-order factors (i.e., exhaustion, cynicism, and inadequacy). In the following years, the SBI has been validated in a few European and North American contexts (Spain–Moyano and Riaño-Hernández, 2013; Turkey–Secer et al., 2013; Italy–Fiorilli et al., 2014; France–Meylan et al., 2015; Switzerland–Gabola et al., 2021; Mexico–Osorio et al., 2019; and United States–May et al., 2020). Although the research mentioned above has led to significant advances in the study of school burnout, more research is warranted to assess the cross-cultural applicability of the SBI, especially in South American countries in which, to the best of our knowledge, no psychometric analyses have been conducted.

The current study aims to examine the psychometric properties of the SBI in a convenience sample of high school Chilean students. For this, we will follow a within-network and between-network construct validity. The first refers to assessing reliability, factor structure, and gender invariance, while the second refers to assessing the extent to which school burnout is associated with theoretically related constructs. In this line, we have selected three constructs that have been shown to play an important role in predicting school burnout. First, study-related emotions (e.g., Burr and Dallaghan, 2019; De la Fuente et al., 2020) are defined as those emotions that emerge in the educational environment and which relate to learning, achievement, and instructional processes that take place in the school (Pekrun et al., 2002). Second, academic psychological capital (e.g., Lupşa and Vîrgã, 2020; Vîrgã et al., 2020), defined as an individual’s positive psychological state of development, characterized by hope, efficacy, resilience, and optimism (Luthans et al., 2015). Third, academic engagement (e.g., Salmela-Aro and Upadyaya, 2012; Sulea et al., 2015), defined as a positive, fulfilling, study-related state of mind characterized by vigor, dedication, and absorption (Schaufeli et al., 2002).

Based on the arguments presented, we hypothesize the following: The SBI will demonstrate acceptable psychometric properties in a Chilean sample of high school students. Also, we expect SBI scores to be direct and significantly related to study-related negative emotions, and inversely and significantly related to study-related positive emotions, academic psychological capital, and academic engagement. Together, the three constructs predict a significant percentage of the variance of school burnout.

## Materials and Methods

### Participants

Nine hundred and seventy-two Chilean high school students (51% girls) in grades 5–12 (i.e., 12–18 years old, M = 14.41, SD = 1.635) participated in the study. They came from three different schools (each of them hosted approximately 550 students) located in two northern regions of the country (Arica y Parinacota and Tarapacá). Of 972 students, 16% were 12 years old, 17% were 13 years old, 17% were 14 years old, 20% were 15 years old, 19% were 16 years old, 8% were 17 years old, and 3% were 18 years old. In addition, 12% correspond to low, 81% to medium, and 7% to high socioeconomic levels.

### Instrument

The SBI is a nine-item self-report scale grouped into three subscales: exhaustion at school (four items), cynicism toward the meaning of school (three items), and sense of inadequacy at school (two items). All items are scored on a six-point scale ranging from 1 (*completely disagree*) to 6 (*completely agree*).

Study–related emotions were measured using 12 items corresponding to two scales of positive (i.e., excited, energetic, inspired, at ease, relaxed, and satisfied) and negative (i.e., angry, anxious, disgusted, fatigued, discouraged, gloomy) emotions from the *Job–related Affective Well–being Scale* (Van Katwyk et al., 2000) adapted to the academic context (Carmona-Halty et al., 2019a). Students answered using a Likert–type with scores from 1 (*never*) to 5 (*always*), reflecting how they feel about their studies (for example, “my studies make me feel at ease” and “my studies make me feel anxious”). Cronbach’s alpha and McDonald’s omega for core study–related positive emotions were 0.736 and 0.734, respectively, and 0.713 and 0.718 for core study–related negative emotions.

Academic psychological capital was measured using the Academic Psychological Capital Questionnaire 12 (APCQ–12; Martínez et al., 2021). The APCQ–12 measure the four dimensions of the psychological capital construct –hope (four items; for example, “Right now I see myself as being pretty successful in my studies”), efficacy (three items; for example, “I feel sure when sharing information about my studies with other people”), resilience (three items; for example, “I usually take the stressful aspects of my studies in stride”), and optimism (two items; for example, “concerning my studies, I’m optimistic about what the future offers me”)– using a Likert–type scale with scores from 1 (*totally disagree*) to 6 (*totally agree*). Cronbach’s alpha and McDonald’s omega for core academic psychological capital were 0.834 and 0.837, respectively.

Academic engagement was measured using the student version of the Utrecht Work Engagement Scale (UWES–9, Schaufeli et al., 2006) adapted to the Chilean context (Carmona-Halty et al., 2019b). The UWES measure the three dimensions of the academic engagement construct – vigor (three items; for example, “When I’m doing my work as a student, I feel bursting with energy”), dedication (three items; for example, “My studies inspire me”), and absorption (three items; for example, “I feel happy when I am studying intensely”)– using a Likert–type scale with scores from 0 (*never*) to 6 (*always*). Cronbach’s alpha and McDonald’s omega for core academic engagement were 0.852 and 0.855, respectively.

### Procedure

The ethics committee of Universidad de Tarapacá approved the research project. The participants voluntarily filled out an online questionnaire during their usual class schedule. For the purposes of this study, a Spanish SBI version was developed following the International Test Commission Guidelines for test translation and adaptation (Muñiz et al., 2013). Prior to general data collection, the SBI items were piloted on a small sample (*n* = 12) of high school students to find difficulties in understanding the translated and adapted version of the inventory. At this stage, no students reported difficulties in understanding the items. Sampling was non-probabilistic by convenience (Ato et al., 2013) and no participant was removed from dataset.

### Statistical Analyses

All data analyses were conducted using Jamovi 1.2 (The Jamovi Project, 2020) and Mplus 8.2 (Muthén and Muthén, 1998). For preliminary analysis, descriptive statistics, normality tests, and gender differences were tested. For reliability analysis, Cronbach’s alpha and McDonald’s omega coefficients, corrected homogeneity index, and test-retest (with a time lag of four-month) index were calculated. For evidence of validity based on the internal structure, a confirmatory factor analysis (CFA) was realized, using the Robust Maximum Likelihood (RML) estimation method. In addition, gender invariance through multi-group CFA and three levels of equivalence were assessed (i.e., configural invariance, metric invariance, and scalar invariance) using changes in CFI and Standard Root Mean Residual (SRMR; Δ < 0.010) as criteria for determining whether measurement invariance was established (Cheung and Rensvold, 2002; Chen, 2007; Dimitrov, 2010). Schreiber (2017) recommendations were used to help evaluate the cut-off and determine model fit for the following indicators: chi-squared (χ^2^) and normed χ^2^, root-mean-squared error of approximation (RMSEA) with a confidence interval (90% CI), comparative fit index (CFI), Tucker-Lewis index (TLI), and SRMR. Finally, to examine criterion validity of SBI, we conducted both correlation and regression analysis between school burnout, study–related (positive and negative) emotions, academic psychological capital (i.e., hope, efficacy, resilience, and optimism), and academic engagement (i.e., vigor, dedication, and absorption).

## Results

Table 1 shows the descriptive statistics for the SBI at item levels. Independent sample *t*-test revealed that there are not statistical significance differences between boys’ (*M* = 3.309, SD = 0.949) and girls’ (*M* = 3.354, SD = 0.960) SBI scores [*t* (970) = 0.728, *p* > 0.05, *d* = 0.047, 95% IC (-0.165,0.076)].

**TABLE 1:** Descriptive information of the school burnout inventory.

	Descriptive statistics				
	Mean (SD)	S	K	Shapiro-Wilk (*p*)	Corrected homogeneity index
1. I feel overwhelmed by my schoolwork.	3.84 (1.37)	–0.16	−0.77	0.93 (> 0.001)	0.39
2. I feel a lack of motivation in my schoolwork and often think of giving up.	3.24 (1.67)	0.12	−1.21	0.90 (> 0.001)	0.56
3. I often have feelings of inadequacy in my schoolwork.	3.11 (1.35)	0.16	−0.58	0.93 (> 0.001)	0.56
4. I often sleep badly because of matters related to my schoolwork.	3.38 (1.74)	0.04	−1.31	0.89 (> 0.001)	0.51
5. I feel that I am losing interest in my schoolwork.	2.89 (1.58)	0.46	−0.92	0.89 (> 0.001)	0.58
6. I’m continually wondering whether my schoolwork has any meaning.	3.57 (1.73)	–0.08	−1.30	0.90 (> 0.001)	0.43
7. I brood over matters related to my schoolwork a lot during my free time.	3.29 (1.42)	0.25	−0.67	0.92 (> 0.001)	0.10
8. I used to have higher expectations of my schoolwork than I do now.	3.66 (1.65)	–0.12	−1.14	0.91(> 0.001)	0.48
9. The pressure of my schoolwork causes me problems in my close relationships with others.	3.02 (1.71)	0.31	−1.20	0.88 (> 0.001)	0.54

*SD = Standard Deviation; S = Skewness; K = Kurtosis.*

### Within-Network Construct Validation

Considering the corrected homogeneity index, it seems convenient to eliminate item seven (see Table 1). Thus, this item was excluded from further analyses. The SBI showed good internal consistency with Cronbach’s alpha and McDonald’s omega indexes of 0.776 and 0.787, respectively. In addition, internal consistency for each dimension were at least sufficient in both exhaustion (ω = 0.65; α = 0.64) and cynicism (ω = 0.70; α = 0.67) dimensions, although slightly lower in the case of inadequacy dimension (ω = 0.52; α = 0.51).

**FIGURE 1:**
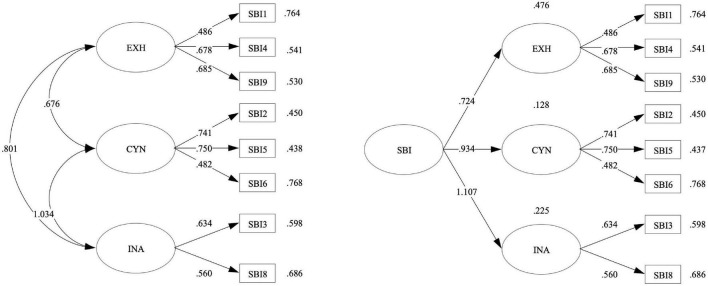
Measurement models of school burnout.

According to previous research on the evidence of the validity of the SBI (e.g., Salmela-Aro et al., 2009a), three models were examined, namely, one that assumes that there is one latent factor underlying all the SBI items (M1), one that proposes three related factors (M2), and one that explains three first-order factors by a second-order factor (M3). The results of the CFA show that M2 and M3 showed an adequate fit (see Table 2 and Figure 1). The multiple-group CFA shows that the differences in the CFI and SRMR across the three invariance models (i.e., configural, metric, and scalar) were lower than .01, which indicates gender invariance (see Table 2). Finally, in terms of test-retest reliability (*n* = 775), the total SBI score shows a correlation of 0.597, while its dimensions reach similar values: exhaustion (*r* = 0.541; *p* < 0.001), cynicism (*r* = 0.548; *p* < 0.001), inadequacy (*r* = 0.501; *p* < 0.001).

**TABLE 2:** Fit indexes for single–group and multiple–group CFA of the school burnout inventory.

	χ^2^	*df*	χ^2^/*df*	RMSEA	90% CI	CFI	TLI	SRMR	CMs	Δ CFI	Δ SRMR
Single–group CFA											
M1 One factor	181.42	20	9.071	0.091	[0.079,0.104]	0.898	0.857	0.049	–	–	–
M2 Three–factors	75.456	17	4.438	0.059	[0.046,0.073]	0.963	0.939	0.030	–	–	–
M3 Second order factor	75.457	17	4.438	0.059	[0.046,0.073]	0.963	0.939	0.030	–	–	–
Multiple–group CFA											
M4 Configural invariance	141.64	34	4.165	0.081	[0.067,0.095]	0.975	0.958	0.027	–	–	–
M5 Metric invariance	141.55	39	3.629	0.074	[0.061,0.087]	0.976	0.965	0.028	M4–M5	0.001	0.001
M6 Scalar invariance	159.50	68	2.345	0.053	[0.042,0.063]	0.978	0.982	0.030	M5–M6	0.003	0.003

*χ2 = Chi-square; df = degree of freedom; RMSEA = Root Mean Square Error of Approximation; CI = 90% Confidence Interval; CFI = Comparative Fit Index; TLI = Tucker-Lewis Index; SRMR = Standardized Root Mean Square Residual; CMs = Comparisons between models.*

### Between-Network Construct Validation

As shown in Table 3, our results showed that school burnout was significant –and in the expected direction– related with all assessed constructs. In addition, they predict a substantial percentage (29.10%) of the school burnout variance measured with the SBI.

**TABLE 3:** Correlations and regression analysis.

	Pearson’s correlation indices				Linear regression indices				
	1	2	3	4	R^2^	F	β	β_std_	*t*
1. School burnout	–				0.291	99.206			
2. Study–related positive emotions	−0.284*	–					0.037	0.026*ns*	0.624
3. Study–related negative emotions	0.485*	−0.275*	–				0.633	0.396*	13.661
4. Academic psychological capital	−0.349*	0.619*	−0.296*	–			–0.157	−0.146*	–3.852
5. Academic engagement	−0.368*	0.742*	−0.352*	0.677*			–0.123	−0.149*	–3.304

*p < 0.001; ns = non-significant.

## Discussion

The current study aims to examine the psychometric properties of the SBI in a convenience sample of high school Chilean students to offer a potential contribution to the school burnout literature.

First, our results suggest that both the three–factors solution and second–order factor of SBI was applicable for high school Chilean students and comparable across gender, which is coherent with previous research (Salmela-Aro et al., 2009a; Moyano and Riaño-Hernández, 2013; Secer et al., 2013; Fiorilli et al., 2014; Meylan et al., 2015; Osorio et al., 2019; May et al., 2020). In addition, reliability analysis, based on Cronbach’s alpha and McDonald’s omega indexes, indicated good internal consistency, whereas the test–retest index shows a moderated relation over a time of four months.

Second, our results suggest that the SBI score relates to other theoretically linked variables to the school burnout construct. In this line, students with high levels of exhaustion, cynicism, and inadequacy, on the one hand, are more likely to experience a high frequency of student–related negative emotions (i.e., angry, anxious, disgusted, fatigued, discouraged, and gloomy), and, on the other hand, a low frequency of study–related positive emotions (i.e., excited, energetic, inspired, at ease, relaxed, and satisfied). In addition, they will also report lower levels of academic, personal resources –measured in terms of hope, efficacy, resilience, and optimism (i.e., psychological capital)– and academic well–being –measured in terms of vigor, dedication, and absorption (i.e., academic engagement)– which is coherent with previous studies (Salmela-Aro and Upadyaya, 2012; Raiziene et al., 2013; Burr and Dallaghan, 2019; Gungor, 2019; Fiorilli et al., 2020; Romano et al., 2021).

Third, our study has some important strengths, such as the large sample used, considering both within-network and between-network approaches, and analyzing the temporal stability of the SBI. However, as a limitation, we can mention that we did not obtain representative data from high school Chilean students; thus, the generalization of the results should be made with caution. Additionally, we use only self–report measures without considering other sources of information (e.g., teachers’reports or grade point average).

Finally, future research can expand our results to other student segments (e.g., undergraduate university students), use samples from different South American countries, and propose cut-off criteria for low, medium, and high levels of school burnout.

## Data Availability Statement

The raw data supporting the conclusions of this article will be made available by the authors, without undue reservation.

## Ethics Statement

The studies involving human participants were reviewed and approved by Comité Ético Científico of the Universidad de Tarapacá. Written informed consent to participate in this study was provided by the participants’ legal guardian/next of kin.

## Author Contributions

All authors contributed equally to the research design and wrote the manuscript.

## Conflict of Interest

The authors declare that the research was conducted in the absence of any commercial or financial relationships that could be construed as a potential conflict of interest.

## Publisher’s Note

All claims expressed in this article are solely those of the authors and do not necessarily represent those of their affiliated organizations, or those of the publisher, the editors and the reviewers. Any product that may be evaluated in this article, or claim that may be made by its manufacturer, is not guaranteed or endorsed by the publisher.
